# Aortic Endograft Infection with *Mycobacterium chimaera* and *Granulicatella adiacens*, Switzerland, 2014 

**DOI:** 10.3201/eid2409.180247

**Published:** 2018-09

**Authors:** Andreas Plate, Thomas A. Kohl, Peter M. Keller, Sabine Majer, Rosamaria Fulchini, Carol Strahm, Cristoforo Medugno, Zoran Rancic, Lars Husmann, Hugo Sax, Stefan Niemann, Barbara Hasse

**Affiliations:** University Hospital Zurich, Zurich, Switzerland (A. Plate, Z. Rancic, L. Husmann, H. Sax, B. Hasse);; Research Center Borstel–Leibniz-Center for Medicine and Biosciences, Borstel, Germany (T.A. Kohl, S. Niemann);; German Center for Infection Research, Borstel (T.A. Kohl, S. Niemann);; National Center for Mycobacteria, University of Zurich, Zurich (P.M. Keller); Institute of Medical Microbiology, University of Zurich, Zurich (P.M. Keller);; Cantonal Hospital Münsterlingen, Münsterlingen, Switzerland (S. Majer);; Cantonal Hospital St. Gallen, St. Gallen, Switzerland (R. Fulchini, C. Strahm);; Cantonal Hospital Frauenfeld, Frauenfeld, Switzerland (C. Medugno)

**Keywords:** Mycobacterium chimaera, Granulicatella adiacens, abdominal aortic endograft infection, cardiac surgery outbreak, whole genome sequencing, tuberculosis and other mycobacteria, nontuberculous mycobacteria, bacteria, Switzerland

## Abstract

We describe an aortic endograft infection caused by *Mycobacterium chimaera* and *Granulicatella adiacens*, successfully treated with prolonged antimicrobial drug therapy after complete explantation of the infected endoprosthesis and extra-anatomical reconstruction. Whole-genome sequencing analysis did not indicate a close relationship to bacterial strains known to cause infections after cardiac surgery.

Aortic endograft infection (AGI) is a serious complication of aortic repair, and treatment involves prolonged antimicrobial drug therapy and complete or partial graft explantation with subsequent in situ or extra-anatomic arterial reconstruction. AGI attributable to nontuberculous mycobacteria (NTM) is a rare condition, and sporadic cases have been described (*1*). *Mycobacterium chimaera* is a slow-growing NTM and a member of the *M. avium* complex. Recent publications show the emergence of disseminated *M. chimaera* infections occurring after open heart surgery (*2*). A field investigation identified contaminated heater–cooler units (HCUs) as the source of infection (*3**,**4*). In addition to valve reconstructions, these cases also involved thoracic aortic grafts. We describe an abdominal AGI caused by *M. chimaera* and *Granulicatella adiacens*. Our aim was to find the source of the *M. chimaera* infection by using whole-genome sequencing (WGS) to compare the patient’s isolate to strains implicated in infections known to occur after cardiac surgery.

## The Study

**Figure 1:**
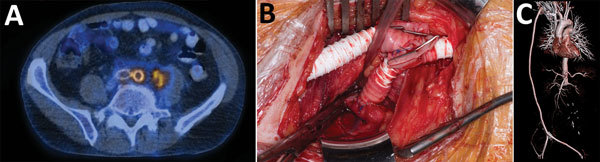
Preoperative, intraoperative, and postoperative images in the case of a patient who received an abdominal aortic endograft and was later diagnosed with *Mycobacterium chimaera* and *Granulicatella adiacens* infection, Switzerland, 2014. A) ^18^Fluorodeoxyglucose positron emission tomography–computed tomography scan at diagnosis indicating a strong, metabolically active (maximum standard uptake value 9.7) aortic endograft infection and an adjacent abscess formation in the iliopsoas in close contact with the left common iliac artery. B) Intraoperative extra-anatomic position of a polytetrafluoroethylene graft through noninfected subcutaneous operative field. C) Satisfactory postoperative result of the axillo-bifemoral bypass on volume-rendered reconstructions of a contrast-enhanced computed tomography.

In March 2014, a formerly healthy 60-year-old man underwent an elective endovascular aortic repair because of an infrarenal aortic aneurysm. In May 2015, the patient sought medical care for low back pain radiating into the left leg. Laboratory examinations showed elevated C-reactive protein (57 mg/L [reference range <5 mg/L]), leukocytosis (14.8 g/L [reference range <9 g/L]), and acute kidney injury (estimated glomerular filtration rate 44 mL/min [reference range >80 mL/min]). A ^18^Fluorodeoxyglucose positron emission tomography–computed tomography (PET–CT) examination indicated an AGI and showed an abscess formation in the iliopsoas muscle in close contact with the left common iliac artery; the intraoperative situs was highly suspicious for AGI, including erosion of the left common iliac artery and a visible endograft. The patient was transferred to the University Hospital Zurich (Zurich, Switzerland) for repeat surgery. The surgical procedure entailed complete endoprosthesis removal, closure of the aortic stump below the renal arteries with polypropylene sutures, and omentum coverage. All tissues were debrided, and treatment included vacuum-assisted open-abdomen treatment. Perfusion of the lower limbs’ arteries was maintained with an axillo-bifemoral reconstruction using a polytetrafluoroethylene graft (Figure 1).

Deep wound cultures obtained during surgical revisions revealed *M. chimaera* (in 3/3 cultures) and *Granulicatella adiacens* (in 4/18 cultures). Histopathologic test results were compatible with mycobacterial infection (Technical Appendix Tables 1, 2). Results of blood cultures and mycobacteriologic blood and sputum cultures remained negative. The patient received a combination therapy containing clarithromycin, rifabutin, ethambutol, and amikacin in the early postoperative phase. After 6 weeks, amikacin was replaced by moxifloxacin. For coverage of *G. adiacens*, amoxicillin was added to the regimen. We treated the patient for a total of 12 months after the extra-anatomic reconstruction. Several PET–CT scans showed a complete metabolic response.

The diagnostic workup in May 2015 revealed an incidental 5-mm small pulmonary nodulus in the right upper lobe, which was observed to be metabolically active in PET–CT. After recovery from the abdominal intervention, the patient underwent wedge resection, and a localized squamous-cell carcinoma of the lung was confirmed. In April 2016, a relapse of his neoplasia occurred. Despite intensified chemotherapy, the patient died in August 2017 because of progressive pulmonary cancer; no autopsy was performed.

We cultured mycobacteriologic samples in BD MGIT tubes (BD, Franklin Lakes, NJ, USA) on Middlebrook 7H11 agar plates (BD) according to previously published methods (*3*). Air and water mycobacterial cultures were performed as suggested by the European Centre for Disease Prevention and Control (*5*).

We analyzed WGS data from the patient’s isolate and strains from published studies (*2*,*6*–*10*) by using a reference mapping approach with the *M. chimaera* DSM-44623 genome (GenBank accession no. NZ_CP015278.1), aided by Burrows-Wheeler Aligner (http://bio-bwa.sourceforge.net), SAMtools (http://samtools.sourceforge.net/cns0.shtml), and GATK (https://software.broadinstitute.org/gatk) software. We combined variant positions to construct a phylogenetic tree with DnaSP 5.0 (http://www.ub.edu/dnasp/index_v5.html), FastTree (http://www.microbesonline.org/fasttree), FigTree (http://tree.bio.ed.ac.uk/software/figtree), and EvolView (http://www.evolgenius.info/evolview) software (online Technical Appendix).

The HCU-related outbreak of disseminated *M. chimaera* infections led us to investigate the hybrid operating room where the patient had undergone his initial surgery (Technical Appendix Figure). The referring hospital did not use HCUs or extracorporeal membrane oxygenation devices. In summer 2015, we obtained water and air samples from the operating room (Table); results were negative for *M. chimaera*.

**Table:** Microbiologic test results of air and water samples from the operating room where an abdominal aortic endograft was performed on a patient later diagnosed with *Mycobacterium chimaera* and *Granulicatella adiacens* infection, Switzerland, 2014

Sample no.	Type	Place of sampling	Result
1	Water	NaCl heater machine	Negative
2	Water	Respirator 1, suction water tank ID 3393	Negative
3	Water	Respirator 1, breathing hose	Negative
4	Water	Respirator 2, suction water tank	Negative
5	Water	Respirator 2, breathing hose	Negative
6	Water	Operating pre-theater, wash basin, siphon	M. intracellulare*
7	Water	Operating pre-theater, wash basin, cold water	Negative
8	Water	Operating pre-theater, wash basin, hot water	Negative
9	Water	Operating pre-theater, sink, siphon	Negative
10	Water	Operating pre-theater, sink, cistern	Negative
11	Water	Operating pre-theater, sink, cold water	*M. paragordonae*
12	Water	Scrub room 2, right side, wash basins 1–3, siphon water	Negative
13	Water	Scrub room 2, right side, wash basins 1–3, after flushing	Negative
14	Water	Scrub room 2, left side, wash basins 4–6, siphon water	Negative
15	Water	Scrub room 2, left side, wash basins 4–6, after flushing	Negative
16	Water	Operating pre-theater, sink, warm water	Negative
17	Air	Air sample 1	Negative
18	Air	Air sample 2	Negative
19	Air	Air sample 3	Negative
20	Air	Air sample 4	Negative
21	Air	Air sample 5	Negative


*Misidentification of *M. chimaera* excluded by partial 16S rDNA sequencing.

**Figure 2:**
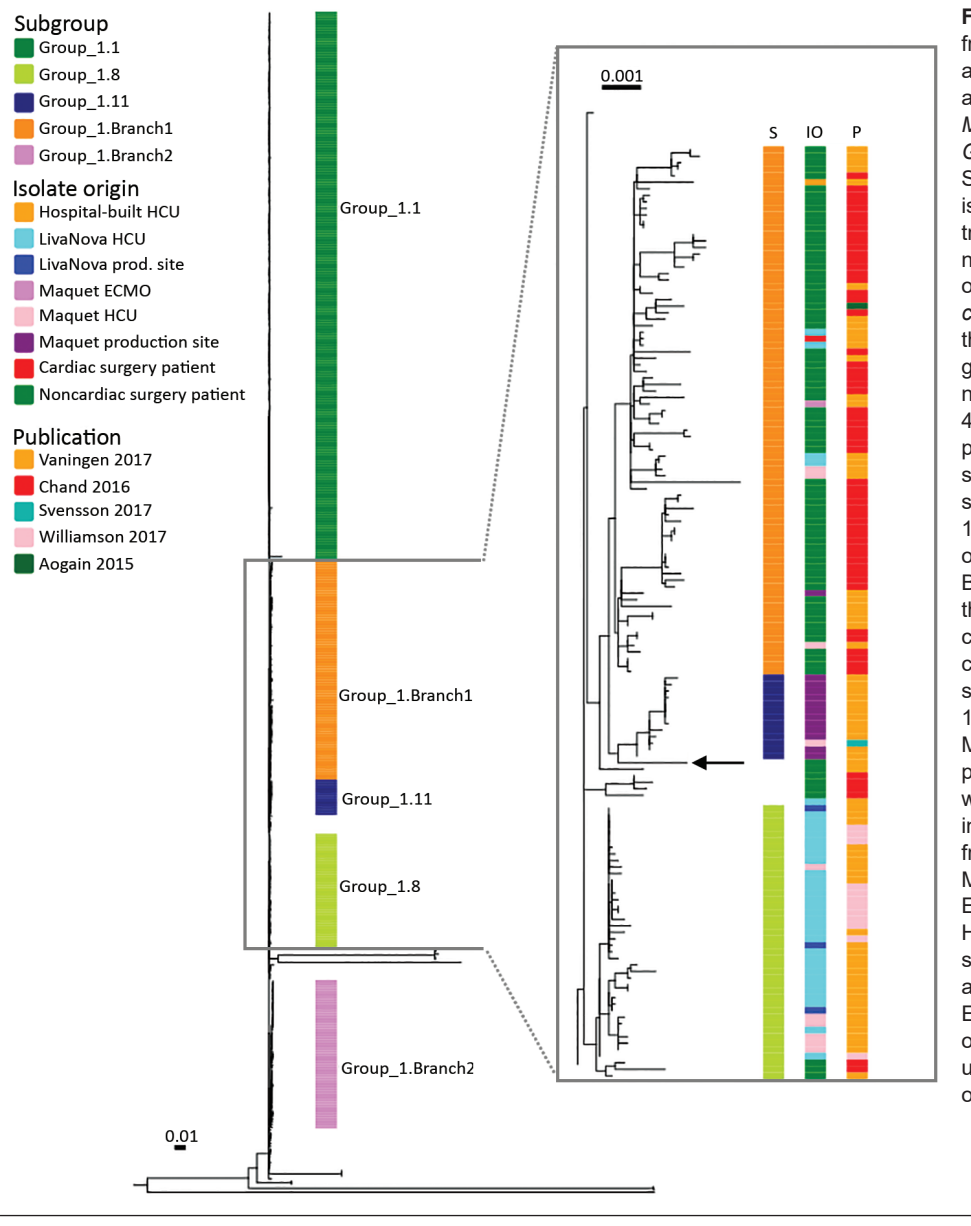
Phylogeny of isolate from case-patient who received an abdominal aortic endograft and was later diagnosed with *Mycobacterium chimaera* and *Granulicatella adiacens* infection, Switzerland, 2014, and comparison isolates. Maximum-likelihood tree was built from 14,192 single-nucleotide polymorphism positions of 437 group 1 *Mycobacterium chimaera* isolates mapped to the DSM-44623 *M. chimaera* genome (GenBank accession no. NZ_CP015278.1). DSM-44623 is shown as a rectangular phylogram with the inferred subgroups indicated. Inset box shows subgroups 1.8, 1.11, and 1.Branch1, annotated with isolate origin and the source publication. Black arrow indicates position of the patient isolate. Group 1.11 consisted mainly of samples collected at the Maquet production site in Rastatt, Germany (n = 12); 1 isolate came from an in-use Maquet HCU. Branch 1 contained primarily strains from patients with pulmonary *M. chimaera* infections (n = 70) and strains from LivaNova HCUs (n = 4), Maquet HCUs (n = 3), Maquet ECMOs (n = 11), a hospital-built HCU (n = 1), Maquet production site (n = 1), and a patient infected after cardiac surgery (n = 1). ECMO, extracorporeal membrane oxygenation; HCU, heater–cooler unit. Scale bars indicate numbers of substitutions per site.

According to a signature single-nucleotide polymorphism–based classification, the patient isolate was similar to the group 1 strains of *M. chimaera* (*2*). We therefore included all group 1 strains with sufficient WGS data from published studies together with the patient isolate in a combined analysis of a total of 437 strains (Figure 2). The patient isolate did not cluster with subgroup 1.1, which represented all but 1 of the reported cases of disseminated *M. chimaera* infections associated with contaminated HCUs. Instead, the patient strain clustered with strains from subgroup 1.11 and branch 1 of group 1 (*2*); however, the patient strain had no close relationship to any other strain included in the comparison.

The endoprosthetic graft (Excluder RMT261214/PXC121200) of our patient was produced by Gore Medical (Newark, DE, USA). The Swiss Agency for Therapeutic Products submitted a medical device report for the implicated graft to the manufacturer.

## Conclusions

We report an endovascular AGI caused by *M. chimaera* and *G. adiacens*, which was successfully treated with extra-anatomic bypass and prolonged antimicrobial therapy. Because of the histopathology results showing focal granulomatous necrotizing inflammation and detection of sparse acid-fast rods in Ziehl Neelsen stain, we outweighed the importance of *M. chimaera* compared with *G. adiacens*.

Patients at risk for NTM infections are elderly patients with preexisting pulmonary conditions or immunocompromised patients. At AGI diagnosis, the localized pulmonic cancer in this patient was in an early stage, and the patient was not known to be immunocompromised. Blood cultures and repeated sputum specimens were negative for mycobacteria, and PET–CT did not reveal any distant foci. Therefore, we considered a hematogenous spread of a localized and naturally acquired infection to be unlikely. Water and air samples from the operating room were negative for *M. chimaera*; thus, local contamination in the operating room was unlikely. When we compared the patient’s isolate with other available *M. chimaera* strains with available WGS data (*2**,**6**–**10*), we observed no association with the cardiac surgery cluster or any other closely related strain in the collection. Because the cardiac surgery cluster originated from *M. chimaera*–contaminated water in medical devices, a contamination of the medical prosthesis at the production site was considered, especially because the poorly soluble polytetrafluoroethylene polymerization is conducted as an emulsion in purified water. However, according to the graft manufacturer, its grafts are produced in a controlled environment, and ethylene oxide gas (EOG) is used for sterilization as recommended by the International Organization for Standardization (standard no. 11135-2007). EOG is widely used because of its good bactericidal activity on many bacterial species and even bacillus spores (*11*). However, studies showing the effect of EOG on mycobacteria are lacking, and cases of NTM infections caused by inadequate implant sterilization have been reported (*12*). As the logical next step in the investigation, testing environmental water samples from the production site or from fresh implants for NTM contamination was proposed. However, because of a paperwork assessment, the company decided not to pursue the case further.

Because our investigation involved a single case of an abdominal AGI caused by *M. chimaera* and *G. adiacens*, it is too early to draw any conclusions. If further infections emerge, investigations into the adequacy of EOG sterilization for arterial implants should be conducted. In this case, the combination of prolonged antimicrobial therapy, graft explantation, and extra-anatomic reconstruction resulted in sustained healing.

Technical Appendix Methods used in whole-genome sequence analysis of *Mycobacterium chimaera* isolates and antimicrobial susceptibility testing results of the *M. chimaera* isolate of the case-patient who received an abdominal aortic endograft and was later diagnosed with *M. chimaera* and *Granulicatella adiacens* infection, Switzerland, 2014.
