# Supplementary material for: Aortic Endograft Infection with Mycobacterium chimaera and Granulicatella adiacens, Switzerland, 2014
Source: Emerg Infect Dis. 2018 Sep;24(9):1700–4. doi: 10.3201/eid2409.180247 (PMC6106423; doi:10.3201/eid2409.180247)
Supplement: Technical Appendix — Methods used in whole-genome sequence analysis of Mycobacterium chimaera isolates and antimicrobial susceptibility testing results of the M. chimaera isolate of the case-patient who received an abdominal aortic endograft and was later diagnosed with M. chimaera and Granulicatella adiacens infection, Switzerland, 2014. [file 18-0247-Techapp-s1.pdf]

Article DOI: https://doi.org/10.3201/eid2409.180247

Aortic Endograft Infection with *Mycobacterium chimaera* and *Granulicatella adiacens*, Switzerland, 2014

Technical Appendix

Methods

Whole-Genome Sequencing Analysis

Whole genome sequence data from the patient's isolate and strains from published studies (*1*–*6*) was analyzed by mapping the reads to the *M. chimaera* DSM-44623 genome (NZ_CP015278.1) using BWA and subsequent refinement of the mappings with the SAMtools (http://samtools.sourceforge.net/cns0.shtml) and GATK toolkits (https://software.broadinstitute.org/gatk). For variant detection, we employed SAMtools and minimum thresholds of a coverage of 4 reads in both forward and reverse orientation, 4 reads calling the allele with a phred score of at least 20, and 75% allele frequency. We inferred a group and subgroup classification as described previously (*1*), with manual curation for mixed populations. For further analysis, we included all 437 datasets from group 1 strains, which reached a mean coverage depth of at least 30 fold, with at least 80% of the reference genome complying with the thresholds stated above. For these, detected variant positions were combined, supplementing the joint list with the respective information from the original mappings where necessary. Single nucleotide polymorphism (SNP) positions fulfilling the thresholds for variant detection in at least 95% of the isolates and covered in all samples, were concatenated to a sequence alignment, excluding SNPs within a window of 12 bp from each other in the same isolate.

From the aligned sequences of concatenated SNPs, we identified homoplasious sites with the recombination detection tool implemented in DNASP v5 and removed these sites from the alignment (496 out of 14,688). From the final set of 14,192 SNP positions, we calculated maximum likelihood trees using FastTree version 2 in the Double precision built, with a general

time reversible (GTR) substitution model, 1,000 resamples and Gamma20 likelihood optimization to account for rate heterogeneity among sites. The consensus tree was rooted with the “midpoint root” option in FigTree (http://tree.bio.ed.ac.uk/software/figtree) and annotated using the EvolView software (http://www.evolgenius.info/evolview).

Technical Appendix Table 1. Overview of surgically derived samples and microbiologic/histopathologic results in the patient

Date	Operation	Specimen	Investigation	Results
22.05.2015	[1,3] Abscess debridement	Deep wound tissue	1/6 Bacteriologic cultures	Coagulase negative Staphylococcus[5]
	[1,4] Coil-embolisation of A. iliaca interna and stent-graft-extension to the left A. iliaca externa		Broad-range PCR	Negative
			Mycobacteriologic culture[2]	*Mycobacterium chimaera.* Time-to-positivity: 9 d
23.05.2015	[1,4] Debridement, NPWT	Deep wound tissue and swab from vascular graft	1/3 Bacteriological cultures	*Granulicatella adiacens.* Time-to-positivity: 5 d
			1/3 Broad-range PCR	*Granulicatella adiacens*
			Mycobacteriologic culture[2]	*Mycobacterium chimaera.* Time-to-positivity: 14 d
			Mycobacterial-genus PCR	*Mycobacterium chimaera*
			Histopathology	M psoas tissue: skeletal muscle and soft tissue with acute inflammation. No evidence of fungi or bacteria. Tissue above graft: necrosis mass. Sparse acid-fast bacilli in Ziehl-Neelsen stain. No evidence of fungi.
29.05.2015	[1,4] Debridement, NPWT	Deep wound tissue and swab from vascular graft	3/3 Bacteriologic cultures	*Granulicatella adiacens*. Time-to-positivity: 3–5 d; MIC Penicillin 0.125 mg/L (S ≤0.25–2 mg/L); MIC Gentamicin 12 mg/L (S ≤2–4 mg/L)
			Mycobacteriologic culture[2]	*Mycobacterium chimaera*. Time-to-positivity: 17 d
			Mycobacterial-genus PCR	*Mycobacterium chimaera*
01.06.2015	[1,4] Endoprosthesis removal. Closure of the aortic stump below the renal arteries. Omentum coverage. NPWT and open abdomen treatment. Axillo-bifemoral reconstruction	Deep wound tissue Vascular graft	6/6 Bacteriologic cultures	Negative
			Mycobacteriologic culture	Not done
			Mycobacterial-genus PCR	*Mycobacterium chimaera*
			Histopathology	Tissue around prosthesis 1: Predominantly necrotic material, old bleeding residuals with adjacent chronic granulomatous inflammation. Tissue around prosthesis 2: Partially calcified soft tissue with chronic granulating, focal granulomatous necrotizing inflammation and detection of sparseacid-fast rods in Ziehl Neelsen stain. Aneurysm sac: Fibrous soft tissue with marginal zone B cell hyperplasia and polytypic plasmocytosis as well as focal chronic granulomatous Necrotizing inflammation. Sparse acid-fast bacilli in Ziehl-Neelsen stain.

Abbreviations: NPWT, Negative pressure wound therapy; MIC; MIC; broad-range PCR, 16S rRNA Gene polymerase chain reaction

Notes: [1] Antimicrobial prophylaxis: At the time of the initial EVAR placement and during the first surgical revisions, the patient received a standard perioperative prophylaxis with Cefazolin 1 g i.v. 20–30 min before the intervention. At the time of the second, third and fourth revision, the patient was under continued antimicrobial therapy and therefore we renounced on perioperative prophylaxis. [2] Only one specimen per surgical procedure was investigated for mycobacteria. [3] Operation performed at the Cantonal Hospital Frauenfeld, Switzerland. [4] Operation performed at the University Hospital Zurich, Switzerland. [5] rated as contamination.

Technical Appendix Table 2. Antimicrobial susceptibility testing of the *Mycobacterium chimaera* patient isolate

Antimicrobial substance	Interpretation
Rifampin	
1 mg/L	S
4 mg/L	S
20 mg/L	S
Rifabutin	
0.1 mg/L	S
0.4 mg/L	S
2 mg/L	S
Amikacin	
1 mg/L	R
4 mg/L	S
20 mg/L	S
Moxifloxacin	
0.5 mg/L	S
2.5 mg/L	S
10 mg/L	S
Clarithromycin	
4 mg/L	S
16 mg/L	S
32 mg/L	S
64 mg/L	S
Ethambutol	
5 mg/L	S
12.5 mg/	S
50 mg/L	S
Linezolid	
1 mg/L	I
4 mg/L	S
16 mg/L	S
Clofazimin	
0.25 mg/L	R
0.5 mg/L	R
1 mg/L	S
4 mg/L	S

Note: In this patient, *M. chimaera* was detected three times by culture with the same AST profile. It is important to note that the terms susceptible (S), intermediate (I), and resistant (R) describe *in-vitro* growth inhibition at a given drug concentration, and are not to be confused with classifications according to clinical breakpoints intended to predict clinical outcome. The intermediate category indicates that the drug concentration examined significantly (>99%), but not completely, inhibits bacterial growth *in-vitro*.

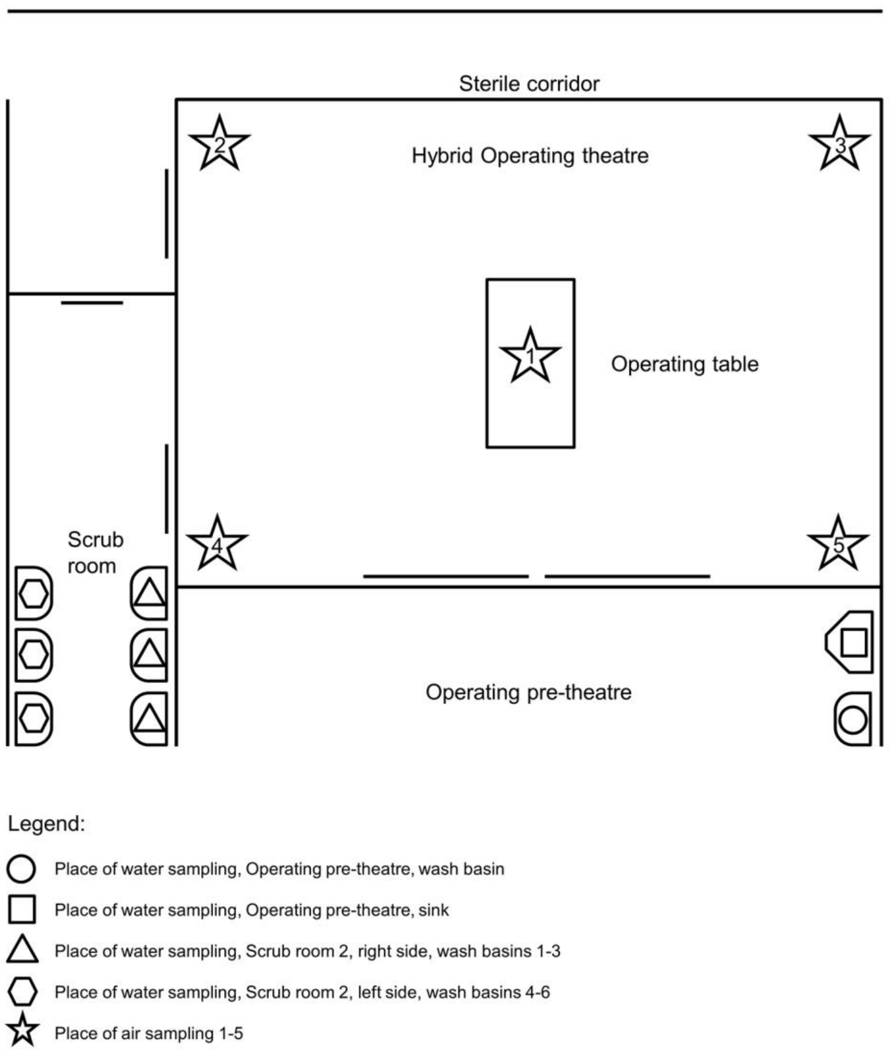

Technical Appendix Figure. Place of sampling at the hybrid operating theater where the endovascular procedure was performed.

References

1. van Ingen J, Kohl TA, Kranzer K, Hasse B, Keller PM, Katarzyna Szafrańska A, et al. Global outbreak of severe *Mycobacterium chimaera* disease after cardiac surgery: a molecular epidemiological study. Lancet Infect Dis. 2017;17:1033–41. PubMed http://dx.doi.org/10.1016/S1473-3099(17)30324-9

2. Chand M, Lamagni T, Kranzer K, Hedge J, Moore G, Parks S, et al. Insidious risk of severe *Mycobacterium chimaera* infection in cardiac surgery patients. Clin Infect Dis. 2017;64:335–42. PubMed http://dx.doi.org/10.1093/cid/ciw754

3. Perkins KM, Lawsin A, Hasan NA, Strong M, Halpin AL, Rodger RR, et al. *Mycobacterium chimaera* contamination of heater-cooler devices used in cardiac surgery—United States. MMWR Morb Mortal Wkly Rep. 2016;65:1117–8. PubMed http://dx.doi.org/10.15585/mmwr.mm6540a6

4. Svensson E, Jensen ET, Rasmussen EM, Folkvardsen DB, Norman A, Lillebaek T. *Mycobacterium chimaera* in heater-cooler units in Denmark related to isolates from the United States and United Kingdom. Emerg Infect Dis. 2017;23:507–9. PubMed http://dx.doi.org/10.3201/eid2303.161941

5. Williamson D, Howden B, Stinear T. *Mycobacterium chimaera* spread from heating and cooling units in heart surgery. N Engl J Med. 2017;376:600–2. PubMed http://dx.doi.org/10.1056/NEJMc1612023

6. Mac Aogáin M, Roycroft E, Raftery P, Mok S, Fitzgibbon M, Rogers TR. Draft genome sequences of three *Mycobacterium chimaera* respiratory isolates. Genome Announc. 2015;3:e01409-15. PubMed http://dx.doi.org/10.1128/genomeA.01409-15
